# Supplementary material for: The genome sequence of the weevil, Pachyrhinus lethierryi (Desbrochers des Loges, 1875) (Coleoptera: Curculionidae)
Source: Wellcome Open Res. 2025 Dec 22;10:687. [Version 1] doi: 10.12688/wellcomeopenres.25212.1 (PMC12895101; doi:10.12688/wellcomeopenres.25212.1)
Supplement: Peer review history

# Reviewer report: Reviewer response for version 1

**DOI:** 10.21956/wellcomeopenres.27788.r145519

**Published:** 2026-02-11

**Authors:** Hong Pang

**Affiliations:** 1 Sun Yat-sen University, Guangzhou, China

I have carefully read through this paper on the genome of *Pachyrhinus lethierryi*. Overall, this work is excellent, with an extremely meticulous approach to genome assembly—one that we can also reference in our future genome assembly projects.

 The authors generated the genome of *Pachyrhinus lethierryi* using third-generation sequencing technologies, namely PacBio HiFi and Hi-C. Haplotype 1 was assembled to the chromosomal level, and assessments including k-mer completeness, QV value, and the BUSCO result (comp.=98.9%) all confirm that this represents a high-quality genome assembly, which lays a solid foundation for subsequent genomic analyses.

Are sufficient details of methods and materials provided to allow replication by others?

Yes

Is the rationale for creating the dataset(s) clearly described?

Yes

Are the datasets clearly presented in a useable and accessible format?

Yes

Are the protocols appropriate and is the work technically sound?

Yes

Reviewer Expertise:

Systematic classification of Coleoptera, biological control, population genetics and resource utilization of ladybird beetles (Coccinellidae).

I confirm that I have read this submission and believe that I have an appropriate level of expertise to confirm that it is of an acceptable scientific standard.

# Reviewer report: Reviewer response for version 1

**DOI:** 10.21956/wellcomeopenres.27788.r143980

**Published:** 2026-01-22

**Authors:** Liang Lü

**Affiliations:** 1 Hebei Normal University, Shijiazhuang, Hebei, China

The manuscript presents the first high‑quality genome for the weevil genus Pachyrhinus. While the study follows a fairly standard format for reporting an NGS assembly, it is strengthened by the inclusion of clear taxonomic, biological, and collection details, as well as high‑quality images that support the reliability of species identification. The workflows for sample preparation, DNA extraction, sequencing, and assembly are consistent with established practices, including those used by the Wellcome Sanger Institute.

 I have two specific points that I would like the authors to address:

 1. BUSCO assessment – Why was OrthoDB version 10 used rather than the more recent version 12? The latter includes a “Coleoptera” lineage dataset, which would provide a more appropriate reference set than “Endopterygota.”

 2. Genome annotation – Given the availability of high‑quality long‑read data, annotation would be both feasible and highly valuable. A benchmarking annotation would significantly enhance the utility of this genome for future users and downstream analyses.

Are sufficient details of methods and materials provided to allow replication by others?

Yes

Is the rationale for creating the dataset(s) clearly described?

Yes

Are the datasets clearly presented in a useable and accessible format?

Yes

Are the protocols appropriate and is the work technically sound?

Yes

Reviewer Expertise:

entomology, nematology, phylogenomics, molecular clock analysis

I confirm that I have read this submission and believe that I have an appropriate level of expertise to confirm that it is of an acceptable scientific standard.
